# The planetary health case for addressing coercion in mental healthcare

**DOI:** 10.3389/fpubh.2025.1673741

**Published:** 2025-09-08

**Authors:** Deborah Oyine Aluh, Jesus David Cortes

**Affiliations:** ^1^Lisbon Institute of Global Mental Health, Lisbon, Portugal; ^2^Comprehensive Health Research Centre (CHRC), NOVA Medical School, NOVA University of Lisbon, Lisbon, Portugal; ^3^Department of Clinical Pharmacy and Pharmacy Management, University of Nigeria Nsukka, Nsukka, Nigeria; ^4^NOVA National School of Public Health, Comprehensive Health Research Centre (CHRC), NOVA University Lisbon, Lisbon, Portugal

**Keywords:** coercion, planetary health, mental healthcare, climate change, epistemic injustice and marginalized groups

## Introduction

Coercion, including involuntary hospitalization, forced medication, isolation, and mechanical restraints, is a controversial practice that remains prevalent in many mental health systems globally. Although these measures are often justified as necessary for safety, growing evidence highlights their association with many adverse outcomes including trauma, mistrust in healthcare systems, and poor long-term recovery (1). While calls to end these practices typically invoke ethical and human rights arguments, this commentary adds a complementary perspective: a planetary health lens that may offer new insights into coercion as an ecological and structural issue potentially affecting both human wellbeing and environmental sustainability.

Planetary health is an interdisciplinary field that explores the consequences of human-induced disruptions to the environment and the subsequent repercussions on human health. From this perspective, we propose that coercion may undermine ecological and social resilience, disproportionately affect marginalized populations (2), and contribute to the healthcare sector's environmental footprint. This commentary presents an exploratory Eco-social framework drawing on Eco-social theory (3), intersectionality (4), and epistemic injustice (5) to examine how environmental stressors, racialized systems, and culturally narrow psychiatric paradigms might converge to contribute to coercion in mental healthcare.

## Environmental determinants, structural inequalities and epistemic injustice

Climate change-related stressors including extreme temperatures, pollution, natural disasters, and displacement disproportionately affect marginalized populations (6), creating mental health vulnerabilities that often intersect with cultural misunderstandings in clinical settings. A growing body of literature suggests associations between climate conditions and mental health outcomes, with emerging evidence pointing to susceptibility in seasonal affective disorder, bipolar disorder, and suicidal behaviors (7–9). Recent research has begun to examine the relationship between ambient temperature and psychiatric presentations more closely. A 24-month prospective study in Turin involving 730 participants found that maximum temperature and humidex index (a measure combining temperature and humidity effects) remained significantly associated with involuntary psychiatric admissions, particularly among those with bipolar disorder diagnoses (10). Similarly, a retrospective analysis of 2,854 patients in Turin between January 2021 and February 2023 identified correlations between seasonal heatwaves (especially June through August) and increased emergency department admissions for psychiatric conditions (*F* = 3.37, *p* = 0.019). The study found associations for severe disorders including bipolar disorder, major depression, personality disorders, and schizophrenia, with effects most pronounced among individuals aged 50–59 years (11). While these findings suggest potential links between environmental stressors and psychiatric crises, the causal mechanisms underlying these associations remain unclear. Environmental toxins have demonstrated neuropsychiatric effects that may influence symptom severity (12), but more research is needed to establish definitive causal pathways.

These environmental vulnerabilities intersect with systemic inequities in concerning ways. When distress manifests in clinical settings, dominant biomedical frameworks might pathologize culturally grounded expressions of suffering, particularly among migrants and racialized communities. This represents what Fricker terms “epistemic injustice” (5), the systematic devaluation of non-Western knowledge and expression. Cultural narratives framing distress as spiritual, collective, or rooted in systemic oppression are dismissed in favor of pharmacological interventions, particularly when service users resist treatments they perceive as harmful or culturally irrelevant (13). This convergence of environmental vulnerability and cultural misinterpretation may create pathways where marginalized groups are disproportionately channeled into coercive care. Mental health systems often lack the conceptual tools to recognize diverse etiologies of distress, potentially leading to misdiagnosis and inappropriate interventions that trigger fear-driven disengagement (14). The result appears to be a cycle where environmental stressors, structural racism, and epistemic exclusion may co-produce conditions that increase the likelihood of coercive practices. While empirical research directly demonstrating these pathways remains limited, it represents an important area for future investigation.

## Environmental impacts of psychiatric treatments

Environmental conditions may influence both the efficacy and safety of psychiatric treatments in ways that could affect care delivery approaches. People with severe mental health conditions show increased vulnerability to heat-related health effects through multiple pathways including thermoregulatory alterations, medication effects, and behavioral symptoms associated with acute psychiatric conditions (15). Psychotropic medications may compound heat-related risks through various mechanisms. Antipsychotics, antidepressants, and medications with antihistaminic or anticholinergic properties can impair heat elimination via parasympathetic pathways. Mood stabilizers such as lithium may experience altered pharmacokinetics during heat exposure, while serotonergic and antipsychotic medications can directly contribute to hyperthermia risk (15). When clinical deterioration occurs under these conditions, it may be misinterpreted as medication non-compliance or primary symptom exacerbation, potentially leading to treatment intensification and, in some cases, coercive interventions.

The physical environment of psychiatric facilities may also influence treatment outcomes and care approaches. A Swedish study examined whether environmental design features could reduce aggressive behavior in psychiatric settings by comparing clinical outcomes between a newer hospital incorporating nine evidence-based stress-reducing design features (including nature-centered design elements) and an older facility with only one such feature. The newer hospital showed statistically significant reductions in compulsory injections (*p* < 0.0027) and a 50% reduction in physical restraints compared to the older facility (16). However, the study did not isolate the effects of specific design elements, such as nature-centered features, precluding causal associations. Nevertheless, nature-based interventions such as access to green spaces and natural light represent potentially valuable non-pharmacological approaches that warrant further investigation. Environmental degradation and increasing urbanization may limit the availability of such therapeutic environmental features, particularly in under-resourced settings.

## Ecological impact of coercive practices

Coercive practices may impose significant environmental burdens. Involuntary admissions result in substantially longer hospital stays, and readmitted patients experience even longer subsequent hospitalizations (17). This cycle not only harms individuals but may inflate the carbon footprint of psychiatric care, contributing to the healthcare sector's estimated 4–5% share of global emissions. Restrictive settings, such as locked wards, isolation rooms, and high-surveillance environments, appear to be energy-intensive since they demand continuous operation of HVAC systems, electronic monitoring, and security infrastructure. These facilities may also limit the use of environmentally therapeutic design, potentially reinforcing a cycle where ecological degradation and coercive practices co-produce harm. Although robust comparative studies are lacking, it seems reasonable to hypothesize that coercive, inpatient-centered models of care could be less environmentally sustainable than community-based alternatives.

## Reimagining mental health care through the lens of planetary health

Redesigning mental healthcare through a planetary health lens requires shifting away from coercive, hospital-centric models toward community-rooted, climate-resilient systems. Some existing models provide preliminary evidence for the feasibility of reducing coercion while potentially enhancing environmental sustainability.

The Open Dialogue (OD) approach, developed in Finland, represents a family-oriented early intervention model that has shown promising outcomes in treating first-episode psychosis. A register-based cohort study examined long-term outcomes over ~19 years, comparing 108 OD patients with 1,763 control patients treated in other Finnish specialized mental health facilities (18). While no differences emerged in annual incidence of first-episode psychosis, diagnostic distributions, or suicide rates between groups, the OD group demonstrated significantly lower rates of hospitalization duration, disability allowances, and neuroleptic medication use throughout the follow-up period. The Trieste Model in Italy offers another example of community-oriented care, having substantially reduced psychiatric bed capacity by repurposing existing community buildings rather than maintaining resource-intensive hospital infrastructure. This approach operates on principles that prioritize patient citizenship, community integration, social inclusion, and preservation of individual freedom and autonomy (19). Other models such as Nigeria's former Aro village system of psychiatry provide additional examples of culturally sensitive, potentially less coercive approaches that integrated care within existing community structures (20). While these models intuitively suggest lower environmental impact through reduced hospital infrastructure, empirical data on their environmental impacts and clinical outcomes remain limited.

## Conclusion and future directions

A system that forcibly detains and medicates those most marginalized, often as a response to distress rooted in systemic and ecological harm, is fundamentally unsustainable, both morally and environmentally. This commentary supports the WHO's call to reduce the carbon footprint of healthcare while strengthening resilience to climate impacts. As the Rockefeller Foundation–Lancet Commission on Planetary Health argues, health professionals must transcend clinical boundaries to support justice and sustainability (21). We propose that this imperative should extend to examining coercive practices in mental healthcare, although we acknowledge that establishing definitive connections requires substantial additional research. Future research should prioritize rigorous methodological approaches including randomized controlled trials of environmental design interventions and comprehensive environmental impact assessments comparing hospital-based vs. community-based care models. Longitudinal studies are needed to clarify causal mechanisms linking climate factors to psychiatric outcomes while controlling for confounding variables. Equally important is empirical testing of proposed pathways connecting environmental vulnerability to coercive care practices, alongside implementation research examining the scalability and cost-effectiveness of alternative care approaches. Planetary health offers a promising conceptual framework for understanding how environmental factors may contribute to coercive practices in mental healthcare. While the associations proposed in this commentary remain largely theoretical, the convergence of climate change urgency and ongoing calls for mental healthcare reform makes this an important area for investigation.
